# Reviewer acknowledgement 2012

**DOI:** 10.1186/2049-6958-8-6

**Published:** 2013-03-19

**Authors:** Fernando De Benedetto, Claudio F Donner, Claudio M Sanguinetti

**Affiliations:** 1 SS Annunziata Hospital, Chieti, Italy; 2 Mondo Medico Clinic, Borgomanero, Italy; 3 Quisisana Clinical Center, Rome, Italy

## Abstract

**Contributing reviewers:**

The editors of *Mutlidisciplinary Respiratory Medicine* would like to thank all of our reviewers who have contributed to the journal in Volume 7 (2012).

## 

Sofia Agelaki

Greece

Nicolino Ambrosino

Italy

Sabina Antoniu

Romania

Lorenzo Appendini

Italy

Eloisa Arbustini

Italy

Tugba Belgemen

Turkey

Salvatore Bellofiore

Italy

Giorgio Bertolotti

Italy

Germano Bettoncelli

Italy

Gian Luca Biscione

Italy

Jean Bourbeau

Canada

Alberto Braghiroli

Italy

Fulvio Braido

Italy

Itzhak Braverman

Israel

Claudio Bruschi

Italy

Stefano Carlone

Italy

Mauro Carone

Italy

Lucio Casali

Italy

Piero Ceriana

Italy

Stefania Cerri

Italy

Marco Colella

Italy

Roberto Colombo

Italy

Sergio C Conte

Italy

Lorenzo Corbetta

Italy

Angelo Corsico

Italy

George Cremona

Italy

Edvardas Danila

Lithuania

Silvestro E d'Anna

Italy

Mehmet Davutoglu

Turkey

Fernando M de Benedictis

Italy

Marino de Rosa

Italy

Antonino Di Stefano

Italy

Hakan Erdem

Turkey

Muriel Fartoukh

France

Evans Fernandez Perez

United States of America

Giovanni Ferrara

Italy

Gabriele Ferretti

Italy

Serhat Findik

Turkey

Maarten Fischer

Netherlands

Giovanni Fontana

Italy

Silvia Forte

Italy

Inez Frerichs

Germany

Giorgio Fumagalli

Italy

Ferretti Gabriele

Italy

Cicchitto Gaetano

Italy

Stefano Gasparini

Italy

Elena Gershtein

Russian Federation

Stefania Giedrys-Kalemba

Poland

Verlato Giovanna

Italy

Cesare Gregoretti

Italy

Markus Henke

Germany

Peter Howard

United Kingdom

Unai Jiménez

Spain

Riitta Kaarteenaho

Finland

Hasan Kahraman

Turkey

Akin Kaya

Turkey

Servet Kayhan

Turkey

Antoine Khalil

France

Mohammad Khan

Canada

Peggy Lai

United States of America

Peter Lange

Denmark

Gennaro Liccardi

Italy

Lichuan Liu

United States of America

Maurizio Luisetti

Italy

Mirco Lusuardi

Italy

Stefano Marinari

Italy

Salvatore Mariotta

Italy

Francesco Massart

Italy

Terence McManus

United Kingdom

Giuseppe Miragliotta

Italy

Maria Alessandra Mirri

Italy

Mehdi Mirsaeidi

United States of America

David Montani

France

Marjukka Myllarniemi

Finland

Stefano Nardini

Italy

Margherita Neri

Italy

Koichi Nishimura

United Kingdom

Yasushi Ohno

Japan

Savas Ozsu

Turkey

Paolo Palange

Italy

Franco Pasqua

Italy

Rogerio Pazetti

Brazil

Giovacchino Pedicelli

Italy

Antonio Pedotti

Italy

Riccardo Pela

Italy

Oronzo Penza

Italy

Isabelle Pham

France

Roberto Piro

Italy

Venerino Poletti

Italy

Francesca Polverino

United States of America

Adriano Massimiliano Priola

Italy

Onofrio Resta

Italy

Thomas Ringbaek

Denmark

Felix C Ringshausen

Germany

Roberto Dal Negro

Italy

Yogesh Saini

United States of America

Alessandro Sanduzzi

Italy

Antonio Sanna

Italy

Francesco Sgambato

Italy

Tania Shakoori

Pakistan

Alan Sihoe

Hong Kong

Michael Sims

United States of America

Matteo Sofia

Italy

David Stav

Israel

Erkan Topkan

Turkey

Roberto Torchio

Italy

Giovanni Viegi

Italy

Alberto Visconti

Italy

Michele Vitacca

Italy

Ozkan Yetkin

Turkey

Zeki Yilmaz

Turkey

Rahizan Zainuldin

Singapore

Pietro Zanon

Italy

Jan Zielinski

Poland

Ali Zubairi

Pakistan

